# Negami: An Augmented Reality App for the Treatment of Spatial Neglect After Stroke

**DOI:** 10.2196/40651

**Published:** 2023-02-27

**Authors:** Britta Stammler, Kathrin Flammer, Thomas Schuster, Marian Lambert, Hans-Otto Karnath

**Affiliations:** 1 Center of Neurology Division of Neuropsychology University of Tübingen Tübingen Germany; 2 Flammer & Gläser UXplain GbR Karlsruhe Germany; 3 XPACE GmbH Pforzheim Germany

**Keywords:** spatial neglect, gamification, augmented reality, visual exploration training, stroke rehabilitation, serious games, rehabilitation, stroke

## Abstract

**Background:**

A widely applied and effective rehabilitation method for patients experiencing spatial neglect after a stroke is “visual exploration training.” Patients improve their ipsilesional bias of attention and orientation by training exploration movements and search strategies toward the contralesional side of space. In this context, gamification can have a positive influence on motivation for treatment and thus on the success of treatment. In contrast to virtual reality applications, treatment enhancements through augmented reality (AR) have not yet been investigated, although they offer some advantages over virtual reality.

**Objective:**

This study aimed to develop an AR-based app (Negami) for the treatment of spatial neglect that combines visual exploration training with active, contralesionally oriented rotation of the eyes, head, and trunk.

**Methods:**

The app inserts a virtual element (origami bird) into the real space surrounding the patient, which the patient explores with the camera of a tablet. Subjective reports from healthy elderly participants (n=10) and patients with spatial neglect after stroke (n=10) who trained with the new Negami app were analyzed. Usability, side effects, and game experience were assessed by various questionnaires.

**Results:**

Training at the highest defined difficulty level was perceived as differently challenging but not as frustrating by the group of healthy elderly participants. The app was rated with high usability, hardly any side effects, high motivation, and entertainment. The group of patients with spatial neglect after stroke consistently evaluated the app positively on the dimensions of motivation, satisfaction, and fun.

**Conclusions:**

The Negami app represents a promising extension by adding AR to traditional exploration training for spatial neglect. Through participants’ natural interaction with the physical surrounding environment during playful tasks, side effects as symptoms of cybersickness are minimized and patients’ motivation appeared to markedly increase. The use of AR in cognitive rehabilitation programs and the treatment of spatial neglect seems promising and should receive further investigation.

## Introduction

Spatial neglect is the dominant disorder after a brain lesion of the human right hemisphere [1,2]. The most common cause of spatial neglect is a large stroke in the right middle cerebral artery area that affects a perisylvian network consisting of the superior/middle temporal cortex and the parietal and ventrolateral frontal cortexes [3]. Affected patients behave as though the left side of their surrounding space no longer exists. The patient’s eyes and head are sustainedly oriented to the side of the brain lesion (ie, mostly to the right side) [4,5]. When searching for objects, the patient’s visual and tactile exploration is shifted to the right side. Accordingly, objects or people located on the left side are ignored and not found. Many therapeutic approaches to spatial neglect thus entail exercises (eg, search training on large projection screens) and activities (eg, reading and copying tasks, picture descriptions) that require increased and active turning of the patients toward the neglected contralesional side [6-9]. In this training, termed “visual exploration training” or “visual scanning training,” exploration movements are improved, and compensatory search strategies are practiced, leading to improvements in neglect behavior in important everyday situations.

A critical component of neurorehabilitation and its success is related to patients’ motivation to train [10]. Improving the latter is one of the main aspects of recent rehabilitation efforts known as “gamification” [11,12]. Gamification describes the application of game design elements in a nongame context to motivate desired behavior. Treatment motivation in stroke rehabilitation can additionally be increased by gamified feedback (ie, giving feedback about the performance in the corresponding task by awarding points) [13].

The principle of gamification is further emphasized when combined with virtual reality (VR) methods. The principle of active exploration training in spatial neglect has already been extended by several VR approaches. Nonimmersive VR procedures, in which a virtual environment is displayed on a monitor with which the patient interacts without being immersed in it, have been successfully used in group studies for therapeutic purposes [14-16]. On the other hand, immersive VR procedures, in which the patient becomes part of a virtual 3D environment surrounding them by means of VR goggles, have also been developed, gearing toward active exploration scenarios. Recent proof-of-concept studies have shown that healthy participants and patients who have had a stroke perceive this technique as highly motivating and enjoyable [17-19]. Moreover, immersive VR applications have already been tested with individual patients with spatial neglect for treatment purposes [20,21].

Another promising way to increase training motivation in poststroke rehabilitation is through augmented reality (AR). AR describes the computer-generated addition of virtual elements to reality. The visual, real world is augmented by virtual images and figures via a video camera of an electronic device such as a tablet. The virtual addition takes place in real time in the sense of a live transmission and thus combines the real and the virtual [22]. Bakker and colleagues [23] developed the first game combining AR elements with visual scanning training in the context of neglect rehabilitation. It consisted of participants searching for virtual images projected onto a wall in the environment of a museum. Design decisions were made based on expert opinion as well as the opinion of patients with spatial neglect. Features to increase extrinsic motivation were rated as the most important design decision.

In this development and feasibility study, the application of an AR technique for the treatment of spatial neglect is further developed. It is based on the observation that visual exploration training seems to be particularly effective when combined with an active rotation of the trunk contralesionally [24]. An attractive extension of this treatment principle is the innovative app named Negami*.* This app is based on the principle that patients are playfully motivated to orient themselves to their neglected side of the real room by (1) following and (2) searching for a virtual element (in this case an origami bird), actively exploring space by turning their gaze, head, and trunk. In developing the game, special consideration was given to the age group to ensure high usability. Our goal was to develop a task that would motivate the patients and that they would enjoy. By eliciting the subjective views of both healthy elderly participants (aged 57 to 89 years) and patients with spatial neglect after stroke regarding our AR-assisted exploratory therapy app, we also aimed to uncover barriers to its use and make recommendations to improve the future development of therapies with AR.

## Methods

### Participants

Ten elderly neurologically healthy individuals with a mean age of 66 (SD 11.9) years, including 5 (50%) females, participated in this study. In addition, we included 10 patients, including 2 (20%) females, who were experiencing spatial neglect and left-sided hemiparesis after a stroke in the neurological right hemisphere —5 (50%) with infarction and 5 (50%) with hemorrhages. They had a mean age of 61.3 (SD 15.2) years and a poststroke interval of 138.4 (SD 192.1) days when they were tested for spatial neglect. More details of the patients are provided in Multimedia Appendix 1.

The inclusion criterion for the patients was the presence of spatial neglect after a stroke. In addition to clinical behavioral observations, diagnostic criteria had to be met in at least 2 of the following 4 neglect tests: the Letter Cancellation Test [25], the Bells Test [26], a Copying Task [27], and a Line Bisection Task [28]. All 4 neglect tests were performed on a Samsung S7+ tablet with screen dimensions of 285 × 185 mm. The severity of spatial neglect in the cancellation tasks was determined by calculating the center of gravity of the target stimuli marked in the search fields, known as the Center of Cancellation (CoC). A CoC value ≥0.08 indicated left-sided spatial neglect [3,29]. The Copying Task consisted of a complex scene consisting of 4 objects (fence, car, house, and tree), wherein points were assigned based on missing details or whole objects. One point was given for a missing detail and 2 for a whole object. The maximum number of points was therefore 8. A score higher than 1 (ie, >12.5% omissions) indicated neglect [27]. In the Line Bisection Task [28], patients were presented with 4 different line lengths eight times each (ie, 32 lines in total). The cut-off value for spatial neglect was an end point weightings bias (EWB) value ≥0.07 [30].

In the group of patients, the mean CoC on the Letter Cancellation Test was 0.42 (SD 0.28), the mean CoC on the Bells Cancellation Task was 0.38 (SD 0.29), the score on the copying test was 4.4 (SD 1.51), and the EWB score on the Line Bisection Task was 0.33 (SD 2.5).

### Ethics Approval

All participants gave their informed consent to participate in the study, which was conducted following the ethical standards of the 1964 Declaration of Helsinki. This study was also approved by the ethics committee of the University Clinic of Tübingen (373/2021B02).

### The Negami App

#### Overview

Negami [31] is a mobile app that enables the treatment of spatial neglect using gamified AR. It is optimized for use on tablets but also works on smartphones. In this study, a 3rd generation Apple iPad Pro 12.9” was used to run the app and conduct the therapy sessions. Negami provides 2 modes (Task A and Task B), which are detailed in the subsequent section. In both tasks, the live video stream from the devices’ back cameras is displayed on the screen. Additionally, a virtual element (origami bird) is added (augmented) as a 3D object that must be followed (Task A) or searched for (Task B) by moving the tablet in 3D space. An overlaying crosshair user interface (UI) element provides visual feedback and guidance for the patient.

#### Technical Background

**Figure 1:**
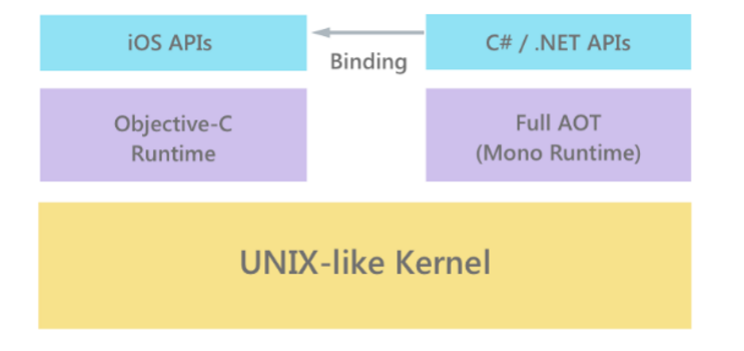
Architecture of an application developed using Xamarin.iOS (left) and Xamarin.Android (right) [33]. AOT: ahead of time; API: application programming interface.

The Negami app can be achieved via the Negami website. The app was implemented in C# programming language using the open-source cross-platform framework Xamarin (Microsoft Corp) [32]. It allows the creation of native Android, iOS, and Windows apps by sharing UI and code across platforms. The Xamarin platform consists of Xamarin.iOS and Xamarin.Android projects, which are implementations of the Mono framework for iOS and Android, respectively. Mono is a cross-platform open-source .NET compatible software framework. Additionally, Xamarin includes the Xamarin.Forms library for the creation of cross-platform user interfaces using XAML (Extensible Application Markup Language). A UI created in Xamarin.Forms is automatically mapped to native controls on each platform during the build process. Xamarin was chosen for the development of the Negami app because it provides native runtime performance, which is important for game-based apps, as well as an abstraction layer over many platform-specific application programming interfaces (APIs), thus increasing development speed. Additionally, Xamarin offers a set of bindings for full access to all device-specific APIs (Figure 1). Apps built with Xamarin are compiled into native packages for each platform. For the Android platform, the written code is compiled down to Intermediate Language (IL), which is then just-in-time compiled to native assembly on app launch. On iOS, the app is fully ahead-of-time compiled into native ARM (Advanced Reduced Instruction Set Computer Machine) assembly code (Figure 1 [33]).

Utilizing Xamarin, the implementation of the Negami app’s entire business logic and user interface could be shared across platforms. The development followed the Model-View-View-Model design pattern and applied other best practices such as the use of an inversion of control container for dependency injection, commands, and event aggregators. Additionally, the app’s functionality was verified using both unit tests and manual testing. To facilitate the data collection process, a data synchronization feature was implemented that regularly syncs the local SQLite database (which enables the app’s offline usage) containing metadata about each patient and app settings with a remote Microsoft SQL (Structured Query Language) database utilizing the Dotmim.Sync library. As the actual data for each completed exercise session were too large to be stored efficiently in a database, a file-based synchronization was implemented using the Azure Blob Storage (Microsoft Corp) service.

Unlike the business logic and UI elements, the AR aspects of the app had to be implemented in a platform-specific way because Xamarin provides no abstraction layer for these APIs. The implementation was only completed for the iOS platform. For this, the Apple ARKit platform [34] was used (through native bindings provided by Xamarin.iOS) to implement all AR-related logic, such as world tracking, placing and rendering of the objects in 3D space, and retrieving the current screen position of the bird. For Android, the Google ARCore library was utilized in the future to implement the same logic.

#### Design

Since the target group consists mainly of elderly people, the design of the app was tailored to their needs to facilitate the most positive user experience. The app was intended to reduce the distance and strangeness that this user group often feels from digital devices. We did not want it to feel like a monotonous, mandatory clinical treatment. Instead, we wanted patients to playfully find the motivation to work on their recovery.

A further aim was to ensure that patients—after discharge from inpatient treatment—can safely use the app to practice on their own or with family members to make further progress. This is achieved through short distances in operation and simple instructions and assistance. The same applies to large control buttons that are easy to reach even with shaky or stiff fingers. We also took into account that patients with spatial neglect ignore information on the contralesional side. For example, this was considered when placing and labeling the exercise buttons. Simple letters like “A” and “B” presented on the right tablet side were used instead of complex icons or text. Moreover, we took into consideration that patients with spatial neglect are often hemiparetic. Instead of holding the tablet from the right and left with both hands, hemiparetic patients can hold it with the unaffected hand from the bottom/center. However, if the tablet is too heavy for the patient to hold in this single-handed position, a loop with Velcro can be attached to the patient’s wrist, which, in turn, is attached to the middle of the back of the tablet to help the patient carry the weight.

In addition to the patients, there is another user group: the therapists. This group of users has different requirements for the app than the patients. Since therapists are often responsible for several patients concurrently, they need to treat patients as efficiently as possible. For this purpose, the app offers the therapist the possibility to easily adjust the corresponding performance level of each patient to their current treatment status. The development of each patient's skills is easily visible to the therapist. Further, the app offers the possibility to create a plan for independent therapy at home. The patient's progress thus is recorded and can be viewed by the therapist at any time, allowing them to make the exercises more difficult or easier depending on the patient's level of ability.

#### Tasks Provided by the Negami App

**Figure 2:**
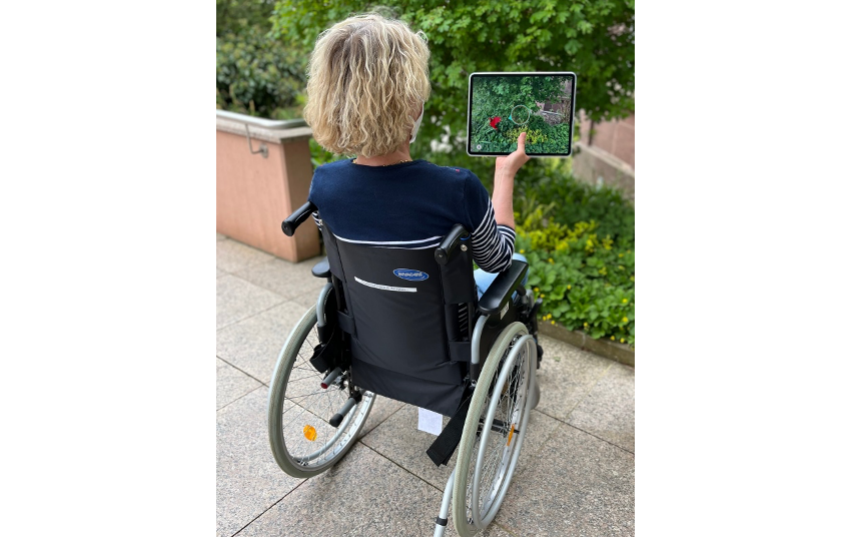
Patient performing tasks of the Negami app. A virtual element (origami bird) was added (augmented) to the video stream of the surroundings produced by the camera of a tablet.

During the typical use of Negami, the participant sits in a chair or a wheelchair. If possible, the tablet is held with both the left and right hands. If the participant is hemiparetic, the tablet can be held from below with one hand positioned in the middle. Using arm movements in combination with trunk rotations, the tablet can then be moved to the left or right through real space (Figure 2). The participants perform 2 different tasks in succession.

**Figure 3:**
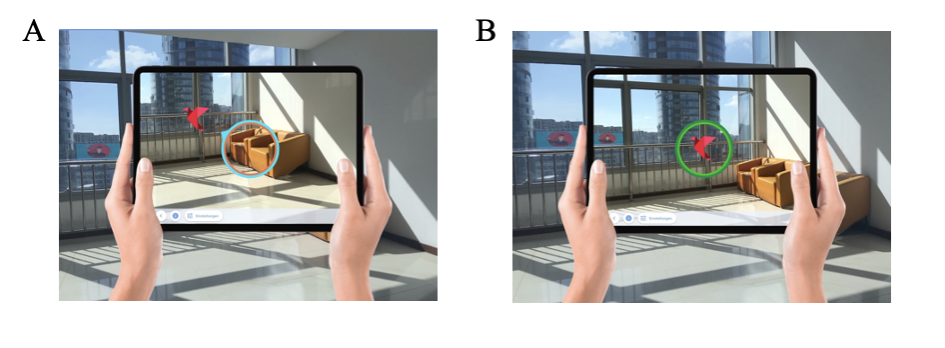
Task A: “follow the bird." The patient has the task to follow the flying origami bird and to keep the bird within the orange/blue circle (A). If the task is successfully solved the circle turns green (B).

The patient’s first task (Task A: “follow the bird”) is to follow the virtual origami bird through real space. The patient's straight-ahead eye/head/body orientation is set as 0. Beginning at set point 0, the bird flies with random sinusoidal movements toward one side of space (in neglect patients toward the contralesional, neglected side). While doing so, the patient sees an orange circle in the center of the screen (Figure 3). The bird should be held in this circle while performing the task. As soon as the patient fails to successfully follow the bird and keep it in the circle during its flight, they are provided with an additional orientation aid. A blue compass needle appears showing the patient in which direction the bird is located (see Figure 3). The task is successfully completed as soon as the bird has finished its trajectory and the bird has been positioned centrally in the orange circle. During the performance, the patient receives auditory feedback. Every time the patient manages to get the bird into the orange circle, a short, bright tone is presented. If the patient manages to keep the bird continuously in the orange circle, the tone is presented every 2 seconds. When the task has been successfully completed, the patient receives feedback auditorily again through a different tone and visually through the change of the color of the circle to green.

At the end of a successful trial, the options “change angle” and “lead to point 0” are provided on the screen. If the patient/therapist wants to repeat the task, they can select “lead to point 0,” and if the difficulty of the task must be varied, the patient/therapist can select “change angle.” The patient/therapist has 3 different difficulty levels to choose from: easy, medium, or difficult (Table 1). The difficulty levels are saved as templates.

**Table 1:** Difficulty levels provided with Task A (“follow the bird”)^a^.

Level	Maximum distance of the bird’s path	Speed of bird movement	Amplitude of bird movement
Easy	up to ±35°	2.3°/sec^b^	6.8°
Medium	up to ±55°	4.1°/sec	8.0°
Difficult	up to ±85°	8.1°/sec	11.3°

^a^Angular degrees are participants’ space coordinates, starting from their straight-ahead eye/head/body orientation, which is set as 0.

^b^sec: second.

**Figure 4:**
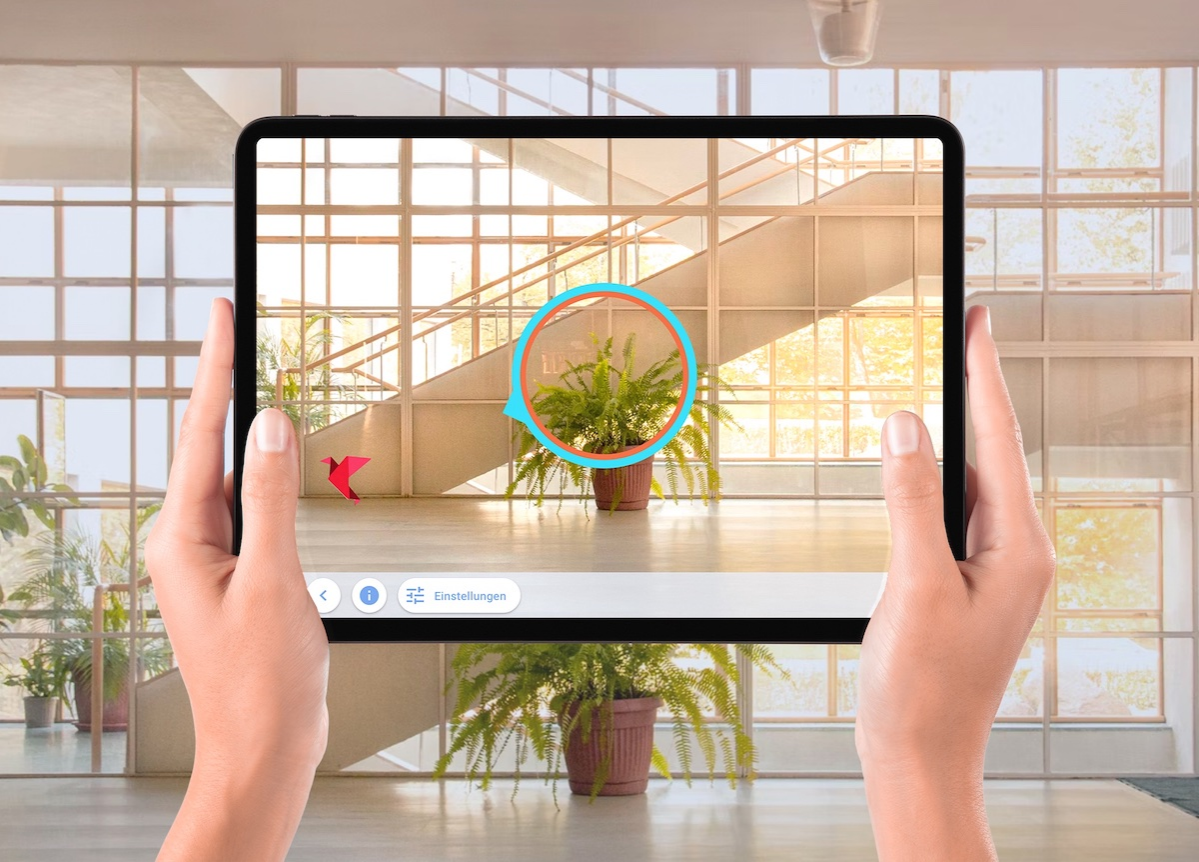
Task B: “find the bird." The patient has to search for the bird that has been hidden by the therapist somewhere in the surrounding room (in this image, at the corner located at the foot of the stairs) and has to transfer it into the orange/blue circle.

In the second task of the Negami app (Task B: “find the bird”), the therapist hides the virtual origami bird in the room surrounding the patient. For this purpose, the patient's straight-ahead eye/head/body orientation again is set as 0. Starting from this location, the therapist hides the bird somewhere on the left/right side of the patient without the patient seeing it. The best way to do this is for the therapist to step behind the patient and hold the tablet above the patient's head in such a way that they cannot see where exactly the tablet is pointing. The area within the bird that can be hidden by the therapist is predefined by the app depending on the chosen difficulty level of the task (Table 2). Once the therapist has positioned the bird in space by clicking on the center of the screen, they return the tablet to the patient's hand. The patient is then instructed to find the bird, and once found, to position it centrally into the orange circle. No time limit is given. Should the patient show difficulties in finding the bird, it is possible to provide the patient with orientation assistance by turning on the blue compass needle (Figure 4). The task is successfully solved when the patient finds the bird and places it in the center of the circle. As with Task A, the circle then turns green (Figure 3), and the patient hears a bright tone signaling the successfully solved task. After successful completion of the task, options “change angle” and “lead to point 0” are provided (see Figure 3) so that the task can be repeated or changed in difficulty.

**Table 2:** Difficulty levels provided with Task B (“find the bird”)^a^.

Level	Extension of the area (along the horizontal dimension of the surroundings) within which the bird can be hidden
Easy	0° to −40° (or 0° to +40°)
Medium	−40° to −75° (or +40° to +75°)
Difficult	−75° to −90° (or +75° to +90°)

^a^Angular degrees are the participants’ space coordinates, starting from their straight-ahead eye/head/body orientation, which is set as 0. Negative values indicate positions to the participant’s left, while positive values indicate positions to the participant’s right.

### Procedure

All neurological patients received training with Negami in their room in the ward of the respective rehabilitation facility. The healthy participants also received the training indoors for comparability. The healthy participants had 1 training session with Negami that lasted 20 to 25 minutes. They completed Task A in the first half and Task B in the second half, each at the highest difficulty level. If patients were not able to use both hands to hold the tablet due to hemiparesis, they were instructed to hold the tablet with the unimpaired hand only by grasping the tablet in the middle from the top or bottom. A video example of a patient working with Negami is shown in Multimedia Appendix 2. To assess feasibility, usability, and game experience, the System Usability Scale (SUS) [35], Simulator Sickness Questionnaire (SSQ) [36], and Perception of Game Training Questionnaire (PGTQ) [37] were administered to the participants directly after the training session.

To assess usability, the participants answered the 10 questions of the SUS through a 5-point Likert scale ranging from 1 (fully disagree) to 5 (fully agree). The mean score was calculated for each participant across all questions. To capture the presence of side effects of simulation technologies, the SSQ was applied. For this purpose, the presence of a series of symptoms grouped under “cybersickness” was tested by a 4-point Likert scale ranging from 0 to 3 (not at all, slightly, moderately, and strongly). The following 15 symptoms were recorded in total: general discomfort, fatigue, headache, eyestrain, difficulty focusing, increased salivation, sweating, nausea, difficulty concentrating, fullness of the head, blurred vision, dizziness, vertigo, stomach awareness, and burping. Again, the mean score was calculated across all items for each patient. Finally, the patients were asked via the PGTQ, which uses a 7-point Likert scale ranging from 1 (fully disagree) to 7 (fully agree), about how motivating, frustrating, challenging, and enjoyable they found the training with Negami to be. Since each of the 4 questions covers a different aspect of the perception of Negami, no mean value was calculated.

The patients with spatial neglect received a 2-week application with 5 training sessions per week. Each session lasted 20 to 25 minutes. As the healthy participants, they completed Task A in the first half and Task B in the second half. The tasks were always initiated for the patients with the easiest difficulty level. The criterion for success in advancing to the next difficulty level was that the task was successfully completed 3 times in a row. A return to a lower difficulty level was indicated when the task was not solved 2 times in a row. Upon completion of the treatment, subjective reports about the use of the Negami app were collected from the patients by using the following three questions: (1) How satisfied were you with the treatment? (2) How motivating did you find the treatment? (3) How much did you enjoy the treatment? The answer options varied from 1 to 6, with 1 being the best result. The patients were further asked whether they would recommend the treatment to others (yes/no) and in an open response format, whether they had any suggestions for improvement.

## Results

### Healthy Participants

**Figure 5:**
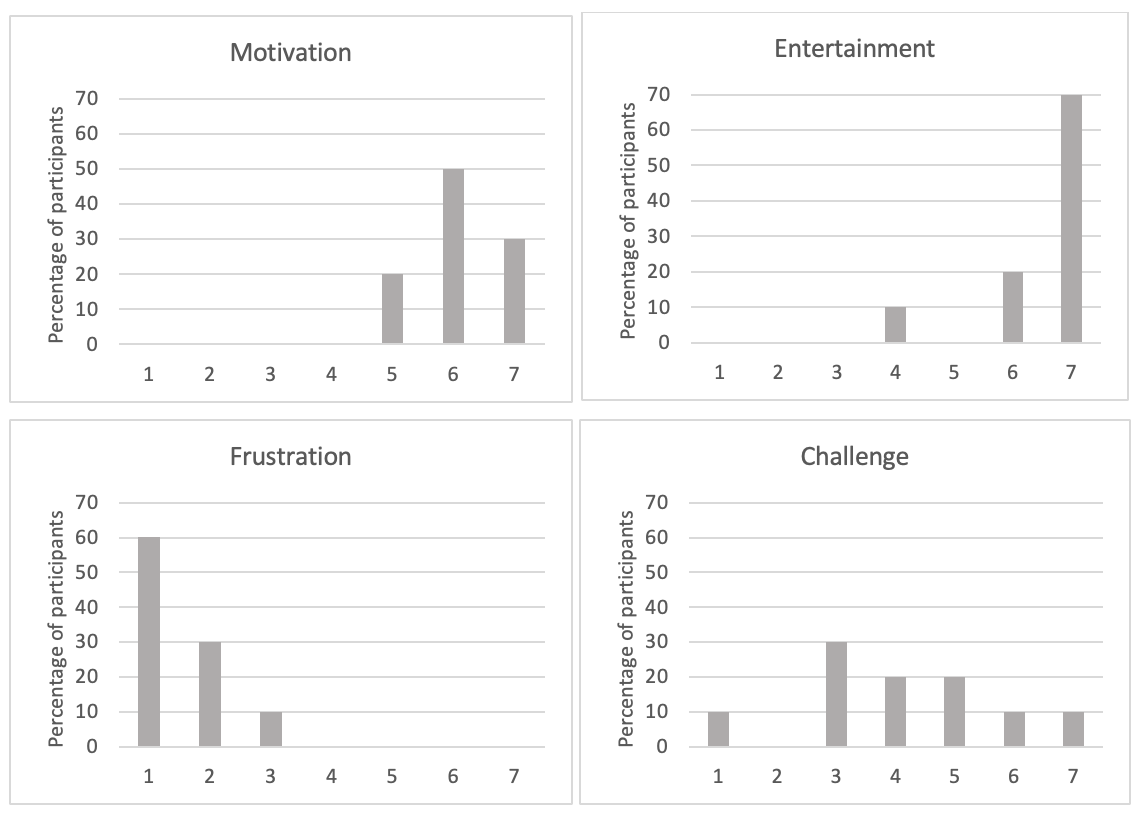
Percentage of healthy, older adults (n=10) evaluating training with the Negami app on the dimensions “motivation,” “frustration,” “challenge,” and “entertainment,” on a 7-point scale (1 = "I fully disagree"; 7 = "I fully agree").

The results of the healthy elderly participants from the SUS on usability resulted in a mean value of 4.3 (SD 0.6), indicating that the usability of the Negami app was rated on average as high to very high. Moreover, there were hardly any side effects detected, as assessed by the SSQ and indicated by a mean score of 0.8 (SD 1.32). More specifically, only 1 patient (10%) reported mild symptoms of fatigue, headache, difficulty focusing, and blurred vision. In addition, 2 (20%) other participants reported mild problems with strained eyes and focusing. None reported moderate or strong side effects. Finally, the PGTQ captured the dimensions of motivation, frustration, challenge, and entertainment during training with Negami*.* The healthy participants’ evaluations on these dimensions are shown in Figure 5. The participants found Negami to be motivating (mean 6.1, SD 0.7) and highly entertaining (mean 6.5, SD 0.9). As indicated by a mean score of 1.5 (SD 0.7), the training was not perceived as frustrating. However, some participants found the training challenging, while others did not (mean 4.1, SD 1.7).

### Neglect Patients

**Figure 6:**
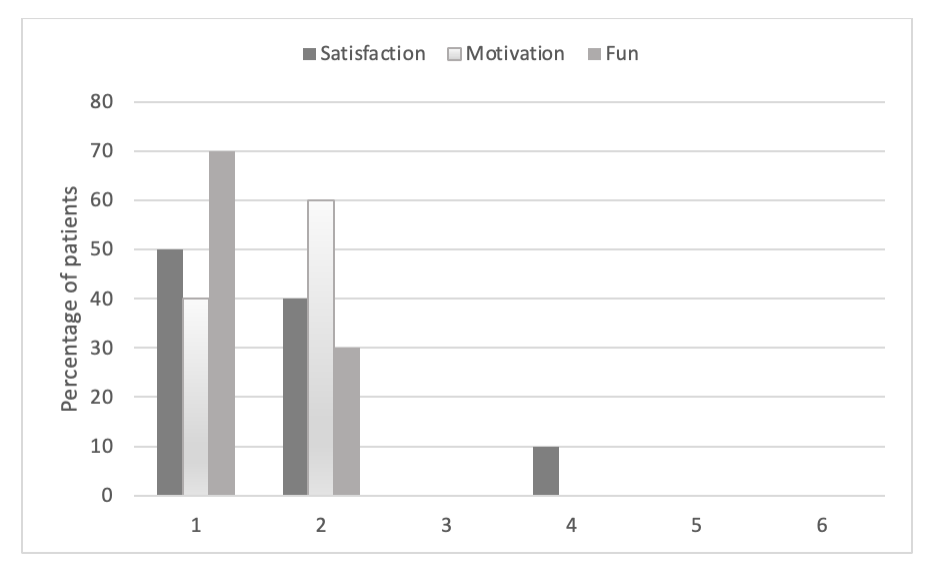
Percentage of stroke patients (n=10) with spatial neglect evaluating training with the Negami app on the dimensions “satisfaction,” “motivation,” and “fun,” on a 6-point scale (1 is the best score; 6 is the lowest score).

An analysis of the reports by the patients regarding the use of the app revealed that all 10 patients recommended treatment with Negami*.* The patients’ evaluation of the app treatment on the dimensions of fun, satisfaction, and motivation are shown in Figure 6. The results demonstrate that all patients evaluated the app treatment on all 3 dimensions with the best 2 scores. Only 1 (10%) of the patients chose a score of 4 on the dimension of satisfaction. Consistent with these very positive ratings by the patients with spatial neglect, the Spearman rank correlation coefficients between the dimensions of satisfaction, motivation, and fun and the severity of spatial neglect (expressed as the mean value of the CoC from the 2 cancellation tasks) were not significant (satisfaction: *r=*.21, motivation: *r*=.57, fun: *r*=.42; all *P*≥.086). Regarding suggestions for improvement in the open response format, 3 (30%) of the 10 patients with hemiparetic neglect reported that they had trouble holding the tablet due to the weight. They had to hold the tablet with 1 hand instead of both. One (10%) patient noted that older patients might be digitally overwhelmed with the treatment. All other patients had no suggestions for improvement.

## Discussion

### Principal Findings

This study aimed to develop and assess the usability, side effects, and game experience of a new rehabilitation app (Negami) for patients with spatial neglect. By using AR, the app was designed to playfully encourage patients to actively explore space by turning their gaze, head, and trunk. Our investigation showed that Negami was perceived as motivating and entertaining by the healthy elderly controls. Training at the highest defined difficulty level was perceived as differently challenging but not as frustrating. The survey further revealed high usability and no to very low side effects such as headache, strained eyes, or blurred vision. Additionally, the second group evaluated in this study (ie, the patients experiencing spatial neglect after a stroke) consistently evaluated the app positively on the dimensions of motivation, satisfaction, and fun. Furthermore, we observed that the app and the tablet could also be used by patients with hemiparesis (by holding the tablet with the unimpaired hand only, grasping the tablet in the middle from the top or bottom). However, 3 (30%) of the 10 patients with hemiparesis complained about the heaviness of the tablet, so the use of even lighter devices seems beneficial.

### Gamification and the Negami App

These positive results give us reason to believe that the new app could become a very useful tool in the treatment of patients with spatial neglect. Ogourtsova and colleagues [38] have shown that patient expectation of performance is the most important factor for acceptance of the use of VR technologies in rehabilitation. Therefore, when defining the 3 different difficulty levels of training with Negami, our focus was on ensuring that the easiest task could be solved relatively quickly even for patients with more severe neurological impairment. This could also be the reason why Negami was so well received by the tested patients who had a stroke. The use of different difficulty levels, as well as auditory and visual feedback on performance during training, are elements that supported the gamification of the Negami app. Gamification for the purpose of rehabilitation has been shown to have positive effects on the patients’ engagement and motivation for their treatment [39]. Another reason for the positive feedback from both patients and healthy participants could be due to our app design in that we paid special attention to the age group, as 75% of patients who have had a stroke are aged over 65 years [40] and are typically not “digital natives” [41]. In implementing short distances in operation, simple instructions, and assistance, we aimed to ensure that this age group feels comfortable in dealing with an electronic device such as a tablet when using the Negami app.

### AR Versus VR Techniques and Telerehabilitation

A third aspect that might have contributed to the positive ratings by the 2 groups tested could be the choice of the AR over VR technique. It is a frequent observation that the use of VR may evoke symptoms that are summarized under the term “cybersickness” [42]. These include symptoms such as drowsiness, headache, balance and coordination problems, nausea, and blurred vision. We observed that such symptoms were absent during the training with Negami. The reason for this could be that the risk of sensory mismatch concerning visual, vestibular, and proprioceptive information, which is considered one of the causes of cybersickness [43], is reduced to a maximum—if not completely avoided—when working with AR. The AR-based Negami app prompts people to move in the real world by actually moving their heads and bodies. The use of the tablet merely adds a virtual object to the real world. In this respect, manipulation is kept to a minimum.

Negami can also offer an advantage over VR games in terms of cost. The development of a VR system is often associated with high costs due to the need for dedicated hardware [42]. Since Negami is available online (see the Negami website), it can be easily downloaded to any available tablet or smartphone. This provides wide availability and ensures that additional costs are avoided. The use of already existing technical hardware also offers immense opportunities regarding remote/telerehabilitation. This is important because, in one-third of patients experiencing spatial neglect in the acute phase of the stroke, the disorder becomes chronic [44]. During their inpatient stay, patients are well cared for in terms of their recovery, but what happens after discharge from inpatient rehabilitation? An answer to that could be remote/telerehabilitation. Easy-to-understand instructions for the tasks in Negami can allow patients to practice independently from home. With online data storage, the therapist has external access to patients’ treatment data and can monitor them remotely.

### Limitations

One limitation of this study is the small sample size. We only could include 10 elderly neurologically healthy individuals and 10 patients experiencing spatial neglect and left-sided hemiparesis after a stroke in the right brain hemisphere. Therefore, testing the Negami app with a larger number of participants in a future study will be useful. It would also be desirable to evaluate the use of the app on the part of the therapists in the future. It should be further mentioned that Negami thus far can only be used on Apple devices. However, it is being further developed with the aim of being able to use on Android devices as well. The further development of all smart devices with different operating systems would also ensure the use of lighter devices, as 3 (30%) of the patients complained about the weight of the tablet. At this point, it should also be mentioned that very severely affected patients, who are not yet able to hold a tablet in the acute phase after a stroke, may not benefit from Negami initially, and other therapeutic approaches should be considered first for them.

### Conclusion

AR represents a promising extension to the traditional “visual exploration training” for spatial neglect. The natural interaction with the environment during playful tasks appears to markedly increase patient motivation. An advantage of AR, as implemented in Negami, over immersive VR-based treatments may be that it minimizes the risk of sensory mismatch concerning visual, vestibular, and proprioceptive information, which is considered one of the causes of cybersickness.

## Appendix

Multimedia Appendix 1 Demographic and clinical data of all 10 patients with right brain damage and spatial neglect.

Multimedia Appendix 2 Patient performing Task B on Negami.

## Abbreviations

API: application programming interface
AR: augmented reality
ARM: Advanced Reduced Instruction Set Computer Machine
CoC: Center of Cancellation PGTQ: Perception of Game Training Questionnaire
EWB: end point weightings bias
IL: Intermediate Language
SQL: Structured Query Language
SSQ: Simulator Sickness Questionnaire
SUS: System Usability Scale
UI: user interface
VR: virtual reality
XAML: Extensible Application Markup Language
