# Supplementary material for: Negami: An Augmented Reality App for the Treatment of Spatial Neglect After Stroke
Source: JMIR Serious Games. 2023 Feb 27;11:e40651. doi: 10.2196/40651 (PMC10012013; doi:10.2196/40651)
Supplement: Multimedia Appendix 1 [file games_v11i1e40651_app1.docx]

**Multimedia Appendix 1: Demographic and clinical data of all 10 right brain damaged patients with spatial neglect**

| Patient | 1 | 2 | 3 | 4 | 5 | 6 | 7 | 8 | 9 | 10 |
| --- | --- | --- | --- | --- | --- | --- | --- | --- | --- | --- |
| Sex (M/F) | M | F | M | F | M | M | M | M | M | M |
| Age (years) | 59 | 72 | 42 | 80 | 43 | 71 | 76 | 65 | 38 | 67 |
| Etiology (infarkt/hemorr.) | inf. | inf. | hem. | hem. | hem. | inf. | inf. | inf. | hem. | hem. |
| Post-stroke interval (days) | 35 | 28 | 658 | 26 | 70 | 85 | 84 | 107 | 234 | 63 |
| Hemiparesis (yes/no) | yes | yes | yes | yes | yes | yes | yes | yes | yes | yes |
| Visual field deficits (yes/no) | no | no | no | no | no | no | no | no | no | no |
| Letters cancellation test (CoC) | 0.69 | 0.15 | 0.25 | 0.67 | 0.73 | 0.03 | 0.01 | 0.54 | 0.61 | 0.53 |
| Bells test (CoC) | 0.9 | 0.28 | 0.24 | 0.74 | 0.33 | 0.12 | 0.10 | 0.52 | 0.10 | 0.54 |
| Copying task (Points) | 5 | 6 | 5 | 4 | 6 | 4 | 1 | 5 | 3 | 6 |
| Line bisection (EWB) | - | 0.04 | 0.15 | 0.2 | 0.3 | 0.2 | 0.42 | 0.76 | 0.19 | 0.69 |

hemorr.=hemorrhage; CoC=Centre of Cancellation [1]; ‘endpoint weightings bias (EWB) [2].

**References**

1. Rorden C, Karnath H-O. A simple measure of neglect severity. Neuropsychologia. 2010;48(9):2758-63. PMID: 20433859. doi: 10.1016/j.neuropsychologia.2010.04.018.
2. McIntosh RD, Ietswaart M, Milner AD. Weight and see: Line bisection in neglect reliably measures the allocation of attention, but not the perception of length. Neuropsychologia. 2017 Nov;106:146-58. PMID: 28923304. doi: 10.1016/j.neuropsychologia.2017.09.014.
